# Isolated perforated jejunal diverticulitis: a case report

**DOI:** 10.1093/jscr/rjaa587

**Published:** 2021-01-31

**Authors:** William P Duggan, Akshaya Ravi, Muhammad A Chaudhry, Felix Ofori-Kuma, Ivan Ivanovski

**Affiliations:** Department of Surgery, Wexford General Hospital, Newtown Rd, Carricklawn, Wexford Y35 Y175, Ireland; Department of Surgery, Wexford General Hospital, Newtown Rd, Carricklawn, Wexford Y35 Y175, Ireland; Department of Surgery, Wexford General Hospital, Newtown Rd, Carricklawn, Wexford Y35 Y175, Ireland; Department of Surgery, Wexford General Hospital, Newtown Rd, Carricklawn, Wexford Y35 Y175, Ireland; Department of Surgery, Wexford General Hospital, Newtown Rd, Carricklawn, Wexford Y35 Y175, Ireland

**Keywords:** Jejunal Diverticulitis, Acute Abdomen, Emergency General Surgery

## Abstract

Jejunal diverticulosis is a rare phenomenon often identified either incidentally on imaging or intra-operatively. Complications of jejunal diverticulosis are associated with high rates of mortality. For this reason, it remains important that this pathology is considered amongst differentials for an acute abdomen. A 78-year old gentleman presented with a short history of generalized lower abdominal pain. Computer tomography scan revealed a large inflammatory abscess relating to a perforated jejunal diverticulum. The patient was taken to theatre where he underwent small bowel resection with primary anastomosis. Early cross sectional imaging is vital to allow early diagnosis and prompt management of this pathology. Small bowel resection with primary anastomosis was associated with an excellent clinical outcome.

## INTRODUCTION

Jejunal diverticulosis is a rare phenomenon with an estimated annual prevalence of between 0.3 and 2.3% [1]. Acquired jejunal diverticulosis often produces few or no symptoms and is generally picked up incidentally on cross-sectional imaging or intra-operatively. Similar to what is observed in colonic diverticulosis, acute inflammation (diverticulitis) can lead to more complicated clinical presentations including; perforation, gastrointestinal bleeding or obstruction. Only a few cases of isolated perforation have previously been reported in English literature [2–5]. Despite the apparent rarity of this pathology, it is associated with a significant mortality rate which was previously reported to range from 21 to 40% [6].

**Figure 1:**
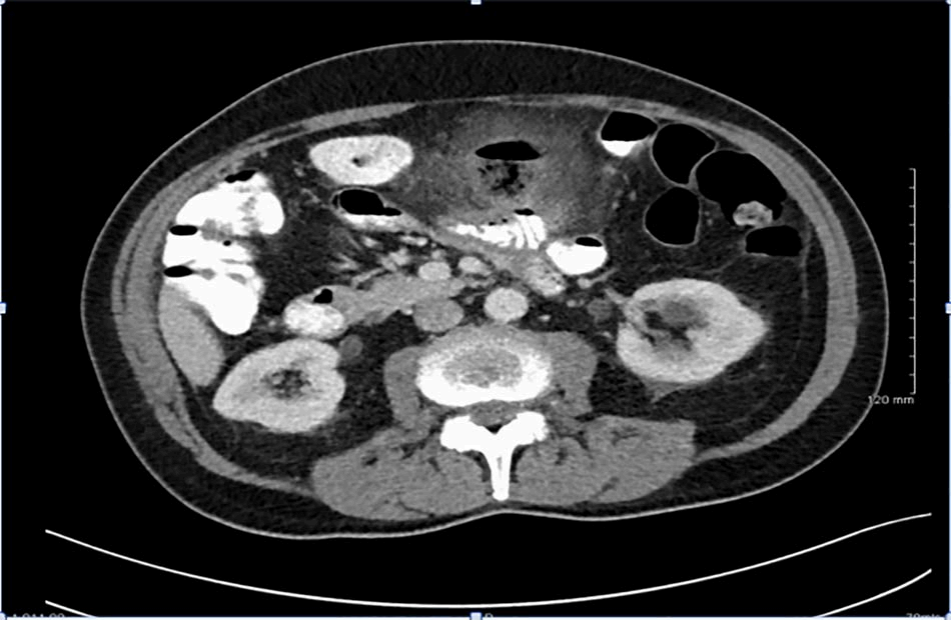
CT findings demonstrating large inflammatory mass related to perforated jejunal diverticulitis.

## CASE REPORT

A 78-year old male presented to the Emergency Department with a 24 h history of sudden onset, generalized lower abdominal pain. Associated symptoms included fever and nausea without vomitus. His past medical history was significant for anal squamous cell carcinoma for which he had been treated successfully with chemo- and radio-therapy 8 years previously. He also previously underwent a laparoscopic cholecystectomy for management of biliary colic. He had no other medical history of note and was taking no regular oral medications.

Clinical examination revealed generalized abdominal tenderness, without objective evidence of peritonism. His temperature was 38.3°C in the emergency room and he was tachycardic at 103. His laboratory markers revealed an elevated white cell count of 10.8 with 92% neutrophilia. His serum C-reactive protein (CRP) was also elevated at 92.3, all other laboratory markers were within normal limits.

A computer tomography (CT) scan of his abdomen and pelvis with oral and intravenous contrast was performed on Day 1 of admission (Fig. 1). CT revealed a large inflammatory mass/abscess cavity containing gas and faecal like content, located centrally in the upper abdomen. The abnormality measured 3.7 x 3.8 x 3.9 cm. There was considerable stranding of surrounding mesentery with thickening of adjacent small bowel and appearances were consistent with a small perforation. His CRP had risen on Days 1 to 243 from 92.3 and he had continued to spike temperatures throughout the day (38.6 and 38.4°C) despite commencement of broad spectrum empirical antibiotic therapy.

**Figure 2:**
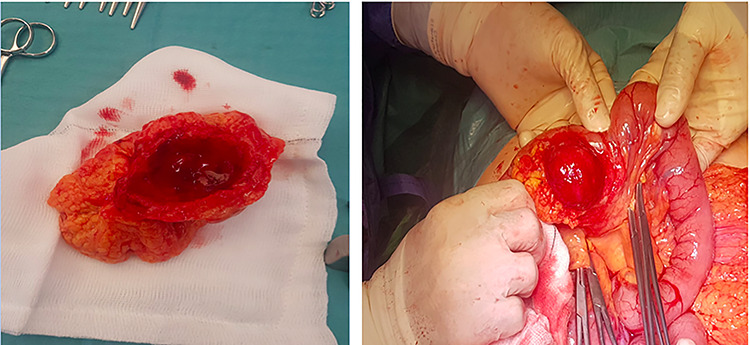
(**a, b**) Intra-operative findings; inflammatory mass located on mesenteric border, relating to perforated jejunal diverticulitis.

The decision was made to take the patient to the operating room where an exploratory laparotomy was performed through an upper midline incision. The inflammatory mass was identified 40 cm from the duodeno-jejunal flexure. The mass was ~4 cm in greatest diameter and located on the mesenteric border of the jejunal segment, covered entirely by omentum (Fig. 2a and b). There was no evidence of a leak or generalized peritonitis. No other small bowel diverticulae were evident on either CT or intra-operatively. A 20-cm segment of jejunum was resected. A hand sewn sided to side primary anastamosis was performed in two layers and the mesenteric window was closed with 2-0 vicryl. Post-operatively, the patients course was uncomplicated. His bowel function returned within 48 h, he was commenced on regular oral diet and was discharged safely on Day 5 post-op.

The histopathology report confirmed the presence of a small bowel diverticulum with associated abscess formation and suppuration.

## DISCUSSION

Diverticulosis is a relatively common disorder, most commonly affecting the colon, however, other sites including the duodenum, jejunum and ileum may also be affected. The causes of small bowel diverticulae specifically are unclear; however, previous studies have postulated that abnormal motor innervation may result in an increase in intraluminal pressure leading to the formation of false diverticulae [7]. Unlike their true counterparts, false diverticulae of the small bowel involve only mucosal and submucosal layers, and most commonly develop on the mesenteric border, which was again the case in this instance. There is an association with male gender, presence of colonic diverticulae, age > 60 and connective tissue disorders [8].

Where the majority of cases of jejunal diverticulosis will be picked up incidentally on imaging or intra-operatively, an estimated 10–30% of cases will present with a complication such as diverticulitis, bleeding, obstruction or perforation [9]. Few cases of isolated jejunal diverticular perforation have previously been reported in the literature. As was the case in this scenario, patients can present with non-specific symptomatology and can deteriorate relatively quickly. As previously mentioned this disease process is associated with a significant mortality rate, in many instances this is owing to a delay in diagnosis [6]. Maintaining a healthy degree of clinical suspicion and securing timely cross-sectional imaging are critical steps in the diagnosis and prompt management of these patients.

Non-operative management of uncomplicated small bowel diverticulitis with antibiotic therapy and bowel rest has previously been reported [10]. However, cases of complicated diverticulitis have largely been managed by operative management with resection and primary anastomosis the predominant approach. Our patient was managed in such a manner and was successfully discharged without the development of complications.

## CONCLUSION

Perforated jejunal diverticulitis is a rare pathology associated with a high mortality rate. We recommend that it is considered within the differential diagnosis of an acute abdomen, particularly in an elderly population. Timely cross-sectional imaging is crucial to allow diagnosis and effective management. Small bowel resection with primary anastomosis was again associated with an excellent outcome.

## CONFLICT OF INTEREST STATEMENT

None declared.
